# A Comprehensive Analysis of the Impact of HIV on HCV Immune Responses and Its Association with Liver Disease Progression in a Unique Plasma Donor Cohort

**DOI:** 10.1371/journal.pone.0158037

**Published:** 2016-07-25

**Authors:** Yong-Hong Zhang, Yan Zhao, Ushani S. Rajapaksa, Tessa M. Lawrence, Yan-Chun Peng, Jinghua Liu, Keyi Xu, Ke Hu, Ling Qin, Ning Liu, Huanqin Sun, Hui-Ping Yan, Emmanouela Repapi, Sarah Rowland-Jones, Robert Thimme, Jane A. McKeating, Tao Dong

**Affiliations:** 1 You’an-Oxford Centre for Clinical Research, Beijing You’an Hospital, Capital Medical University, Beijing, China; 2 CAMS- Oxford University joint International Centre for Translational Immunology, Nuffield and Radcliffe departments of Medicine, University of Oxford, Oxford, United Kingdom; 3 Beijing Key Laboratory for Biomarkers in Infection Related Diseases (BZ0373), Beijing, China; 4 MRC Human Immunology Unit, Weatherall Institute of Molecular Medicine, University of Oxford, Oxford, United Kingdom; 5 Viral Hepatitis Laboratory, Centre for Human Virology, College of Medical and Dental Sciences, University of Birmingham, United Kingdom; 6 Beijing Di’Tan Hospital, Capital Medical University, Beijing, China; 7 Computational Biology Research Group, Weatherall Institute of Molecular Medicine, University of Oxford, Oxford, United Kingdom; 8 Nuffield Department of Medicine, University of Oxford, Oxford, United Kingdom; 9 University Hospital Freiburg, Department of Medicine II, Hugstetter Str 55, 79106, Freiburg, Germany; 10 Department of Microbiology, Faculty of Medicine, University of Colombo, Colombo, Sri Lanka; University of Montreal Hospital Research Center (CRCHUM), CANADA

## Abstract

**Objective:**

Human Immunodeficiency Virus (HIV) and Hepatitis C virus (HCV) co-infection is recognized as a major cause of morbidity and mortality among HIV-1 infected patients. Our understanding of the impact of HIV infection on HCV specific immune responses and liver disease outcome is limited by the heterogeneous study populations with genetically diverse infecting viruses, varying duration of infection and anti-viral treatment.

**Methods:**

Viral-specific immune responses in a cohort of 151 HCV mono- and HIV co-infected former plasma donors infected with a narrow source of virus were studied. HCV and HIV specific T cell responses were correlated with clinical data.

**Results:**

HIV-1 accelerated liver disease progression and decreased HCV specific T cell immunity. The magnitude of HCV specific T cell responses inversely correlated with lower HCV RNA load and reduced liver injury as assessed by non-invasive markers of liver fibrosis. HIV co-infection reduced the frequency of HCV specific CD4^+^ T cells with no detectable effect on CD8^+^ T cells or neutralizing antibody levels.

**Conclusion:**

Our study highlights the impact of HIV co-infection on HCV specific CD4+ T cell responses in a unique cohort of patients for both HCV and HIV and suggests a crucial role for these cells in controlling chronic HCV replication and liver disease progression.

## Introduction

HCV co-infection is recognized as a major cause of morbidity and mortality among HIV-1 infected patients [1]. HIV-1 co-infection is associated with increased HCV load and accelerated rates of liver disease progression [2, 3]. HCV is now the leading cause of death in HIV co-infected subjects, with end stage liver disease accounting for up to 50% of deaths [4, 5].

The importance of viral-specific T cell responses in the early control of HIV and HCV and resolution of HCV infection are well documented [6]. Similarly viral specific T cell responses in chronic HIV and AIDS are well studied compared to HCV. Vigorous HCV specific CD4^+^ and CD8^+^ T cell responses are detectable in acute infection and their appearance associates with the control of viraemia [7]. The central role of T cells in defining the outcome of HCV infection was clearly demonstrated in the chimpanzee model, where depletion of CD4^+^ and CD8^+^ memory T cells led to viral persistence and prolonged viraemia, respectively [8, 9]. Furthermore, vaccine induced multifunctional T cells associated with early control of viral replication in chimpanzees [10, 11]. However, the chimpanzee is not suitable to study the relationship between HCV specific immune responses and disease progression or the impact of HIV co-infection.

The role of HCV specific T cells in HIV co-infection is unclear [12, 13]. HCV specific CD8^+^ T cell frequencies were reported to be lower compared to HIV specific CD8^+^ T cell responses in HIV/HCV co-infected patients [14]. Moreover, the same study suggested that HIV and HCV specific CD8^+^ T cells have distinct phenotypes [14]. However, interpretation of immune studies of HIV/HCV co-infected subjects can be difficult and compromised due to the heterogeneity of the study populations, where patients can be infected through different routes (injecting drug users, men who have sex with men); long term drug treatment for both virus, derive from diverse ethnicities; show different clinical stages of HIV or HCV infection and be infected with genetically diverse viral strains.

To overcome these limitations, we studied a unique population based outbreak of HIV-1/HCV co-infection that occurred in a rural community in central China following paid plasma donation scheme within a narrow period between 1993 and 1995 [15]. HIV-1 and HCV transmission among paid plasma donors in China are believed to have occurred as a result of contaminated blood collection equipment or pooled red cells being returned to donors [16]. Thus, all subjects in our cohort (SM cohort) were infected from a narrow genetic source of HIV-1 and HCV strains circulating over a short period of time [17]. These subjects have been concurrently infected for over two decades and many subjects were classified as HIV-1 slow progressors not requiring HAART [17]. While most of the HIV infected patients were HAART naïve some received HAART for a short duration (less than two years at the time of last sample collection date). Furthermore, HCV infected subjects were not treated with interferon or direct acting antiviral agents. Thus, this cohort provides a unique setting to study the natural history of concurrent HIV-HCV co-infection and to assess the impact on viral specific immune responses and disease progression. To our knowledge the homogeneity of this cohort and treatment naïve nature for HCV are what distinguishes this study from other reports.

## Materials and Methods

### Study population

Samples were collected from identified former plasma donors with HCV mono-infection and chronic HIV/HCV co-infection, living in a small village in Henan province, China. All individuals provided written informed consent. A total of 151 patients were recruited into this study, none were treated with HAART before 2007. The patients who went on HAART, received the treatment for no more than 2 years at the time of recruitment to study. None of the patients were treated for HCV.

### Laboratory evaluations

Patients had standard laboratory assessments performed by trained research staff and clinicians at Beijing You’an Hospital clinical laboratories, including a complete blood cell count, serum bio-chemical profiles, alanine aminotransferase(ALT), alkaline phosphatase(ALP) and gamma glutamyl transferase(γ-GT) levels, CD4 cell count, and plasma HIV-RNA level. HCV antibody testing was performed using Elecsys Anti-HCV (Roche Diagnostics GmbH, Germany).

### Viral RNA load and genotyping

HIV RNA in plasma was detected via bDNA (VERSANT HIV-1 RNA version 3.0 bDNA Assay, Siemens Diagnostics) with a detection limit of 50 HIV-1 RNA copies/ml. HCV RNA was measured in serum using the qualitative Roche COBAS Amplicor assay [version 2.0; Roche Molecular Systems (Branchburg, NJ, USA) lower limit of detection 50 IU/ml]. HCV genotypes were determined by PCR- PCR-reverse hybridization assay (Da An Gene, Guangzhou, China) in serum samples of all patients who are positive with HCV RNA.

### Transient Elastography

Transient elastography (TE) is a non-invasive procedure that measures the velocity of the ultrasound pulse wave that is transmitted to the liver. The velocity correlates to the liver stiffness (LS), which in turn reflects the degree of fibrosis [18–20]. TE was performed with a FibroScan^®^ device (EchoSens^®^—Paris, France) by experienced physicians. In each patient, 10 valid measurements were performed, after which a median value of LS was obtained, measured in kilopascals (kPa). Only patients in which LS measurements by TE had a success rate (the number of successful acquisitions/the total number of acquisitions) of at least 60%, with an interquartile range (IQR) < 30%, were included in our study. IQR is the difference between the 75th percentile and the 25th percentile, essentially the range of the middle 50% of the data.

### Synthetic peptides for T-cell analysis

A total of 479 overlapping peptides (18-mers overlapping by 10 amino acids) spanning the complete amino acid sequence of the HCV core, p7, ARFP, E1, E2 and NS2, NS3, NS4 and NS5 protein derived from HCV-1 genotype 1b (BEI Resources) and 2a (Sigma-Aldrich Co) were utilized. Peptides were dissolved in DMSO (Sigma, Haverhill, Suffolk, UK) at 100mg/ml and diluted with RPMI 1640 (20 mg/ml, long-term stock stored at -80°C) before being individually filtered and combined into 15 pools with 7–50 peptides per pool (S1 Table).

### Ex vivo IFNγ ELISPOT assay

200,000 PBMCs with 8μg/ml peptide were used in a standard Human IFNγ ELISPOT assay as described elsewhere [21]. To quantify antigen-specific responses, mean spots of the control wells were subtracted from the positive wells and results expressed as SFU/10^6^ PBMCs. Responses were considered positive if results were at least 3 times the mean of the quadruplicate negative control wells and over 25SFU/10^6 PBMCs. If background wells had more than 30SFU/10^6 PBMCs or positive control wells (phytohemagglutinin or FEC stimulation) were negative, the assay was excluded from further analysis. The 1b and 2a HCV subtype peptides inducing T cell response magnitude was comparable (S1 Fig). HCV subtype 1b peptides were used in T cell response studies.

### Depletion of CD8+ T and CD4+ T cells

CD8^+^ T and CD4^+^ T cells were depleted according to manufacturers’ instruction (Dynabeads M-450; Invitrogen, Dynal, Oslo,Norway). This method has been validated and widely used [21]. The frequency of CD8^+^ T cells and CD4^+^ T cells were all less than 1% after depletion. The CD4^+^ and CD8^+^ dependency of individual responses were further confirmed by *in-vitro* expansion of HCV antigen specific T cells and intracellular IFNγ co-staining with CD4 or CD8 antibody.

### Antibodies and multicolor Flow Cytometric analysis

The following antibodies were used. CD4 (conjugated to eFluor-450, clone OKT4, Company eBioscience) and CD8 (conjugated to PE Cy7, clone SK1, Company BD), LIVE/DEAD cell viability stain (aqua, Thermo Fisher Scientific) IFN-γ (conjugated to AF488, clone B27, BD), CD3 (conjugated to APC H7, clone SK7, company BD). Cells were stained for surface markers as manufacturer’s instructions. Briefly, cells were washed in PBS, pelleted by centrifugation at 1500rpm for 5 minutes and the supernatant removed completely before incubating with the pre-titrated amounts of antibodies. The cells were incubated on ice for 20 minutes and washed with 2ml of FACS wash buffer (PBS+2%Fetal Calf Serum+0.01% sodium azide). Cells allocated for intracellular staining were permeabilized with Perm/fix (BD, Oxford, UK) for 15 min and washed twice with 1× perm/washing buffer (BD). Cells were then labeled with IFN-γ. Cells were subsequently washed twice with 1× perm/washing buffer and fixed in BD cellfix (BD). Cell events were acquired on a nine-color CyAn Cytometer (Dako, Ely, UK), and data files were analyzed using FlowJo software. Single-color samples were run for compensation, and fluorescence minus one (FMO) control samples were also applied to determine positive and negative populations, as well as channel spillover. See S2 Fig for gating strategy.

### Generation of Antigen-Specific T-Cell Lines and enrichment

Antigen-specific short-term T-cell lines (STLs) were generated as previously described [22]. Briefly, PBMCs were pulsed with NS3 peptide pool for 90 minutes and cultured in 96-well plates in RPMI1640 supplemented with 10% vol/vol human AB serum (National Blood Service). Interleukin-2 (PeproTech EC) was added to a final concentration of 200U/ml on day 3. STLs were restimulated with peptides every 10–15 days.

For enrichment, cell line was stimulated with NS3 peptide pool for 3–4 hours followed by labelling with human IFNγ capture kit (Miltenyi Biotec). High IFNγ producers were sorted on a MoFlow cytometer (DakoCytomation, gating strategy see S2 Fig). Sorted IFNγ+ cells were expanded using phytohemagglutin-treated irradiated allogeneic PBMCs and cultured with IL-2 100U/ml, as described elsewhere [22]. IFNγ negative population from each cell line was also sorted and expanded, as a negative control.

### Antiviral activity of T cells

Enriched HCV NS3-specific CD4^+^ and CD8^+^ T cells were pulsed by peptide at a concentration of 5ug/ml, 0.5ug/ml, 0.05ug/ml and 0.005ug/ml for 1 hour and cultured at a density of 1X10^6^ cells/ml at 37°C, 5%CO_2_ for 24 hours, before collecting supernatant and storing at -80°C. Fifty thousand (50,000) JFH-1 Huh7.5 replicon cells were plated per well of a 48-well plate and cultured with 125ul of Dulbecco's modified Eagle's medium supplemented with 10% fetal bovine serum and 125ul of supernatant collected from peptide-pulsed T cells. HCV replication was assessed by measuring the luciferase activity of the replicon cells after 48 hours. The suppression of HCV replication by T cells was calculated by the percentage of reduction of HCV replication at the presence of supernatant compared to the replication without supernatant.

### HCV neutralizing antibody assay

HCVpp were produced as previously reported [23]. Huh-7.5 hepatoma cells (Charles Rice, The Rockefeller University, New York, NY) were seeded in 96 well plates at 1.1x10^5 cells/ml. The following day serum from patients not exposed to HAART was diluted 1:1000 and incubated with HCVpp strain H77 at 37°C for 1 hour and allowed to infect target cells. Seventy-two hours post infection, cells were lysed and luciferase activity (relative lights units [RLU]) measured to assess infectivity. The percent neutralization was determined by subtracting the mean of an empty vector from the HCVpp value and calculated relative to uninfected patient serum.

### Statistical Analysis

Factors considered in analysis included presence of HCV and/or HIV infection, past and current HAART treatment, serum ALT, ALP, γGT, albumin:globulin ratio, HIV-RNA level, CD4^+^ cell count, LS, antibody mediated viral suppression, and CD8^+^/CD4^+^ responses (SFU) etc. Mann-Whitney test was used for comparison of T cell response magnitude, virus load and liver stiffness, and Kruskal-Wallis test followed with Dunn’s multiple comparisons test to compare neutralisation data. Linear regression was used for comparison of T cell response magnitude induced by 2a and 1b HCV peptides to same sample. One-way ANOVA with Bonferroni post test correction was carried out for group mean comparisons. Statistical analysis and graphs were analysed using Prism 6 (Graphpad software Inc.)

### Ethics

This study was approved by the local ethics committee of Beijing Youan Hospital and the University of Oxford Tropical Ethics Committee (OXTREC 004–05).

## Results

### Clinical characteristics of SM Cohort

A total of 151 HCV infected subjects were recruited to our study and divided into three groups, stratified by HIV-1 infection status: Group 1 (HCV), group 2 (HIV/HCV) and group 3 (HIVt/HCV)). All these patients participated in a plasma donation scheme from 1993–1995 and considered were infected with a narrow genetic source of HIV-1 and HCV strains circulating over a short period of time [17]. They have been concurrently infected over 15 years at the time of sample collection (between 2010–2011) with HIV-1 and HCV. Each group was further classified with respect to their ongoing HCV infection (chronic [HCVc] versus resolved [HCVr]) and HAART treatment for less than two years (HIVt) for study purposes at the time of recruitment. Demographic and clinical data is listed in Table 1. HCV genotype could not be determined in a small proportion of subjects. As expected, significantly higher CD4^+^ counts were observed in HCV mono-infected (Group 1) compared to HIV co-infected persons at the time of recruitment (Group 2 and 3) (*p<0001)*.

**Table 1:** Patient characteristics of 151 individuals infected with HCV and /or HIV. Patients in Group 1, 2 and 3 are considered infected with HIV/HCV for over 15 years before sampling. Group HCVc was mono-infected, HCV chronic. Group HCVr was mono-infected, HCV spontaneously resolved. Group HIV/HCVc was HIV-1+, HCV chronic, HAART naïve. Group HIV/HCVr was HIV-1+, HCV spontaneously resolved, HAART naïve. Group HIVt/HCVc was HIV-1+, HCV chronic, HAART treated. Group HIVt/HCVr was HIV-1+, HCV spontaneously resolved, HAART naïve. ALT, Alanine transaminase; Tbil, Total bilirubin; ALP, Alkaline phosphatase: γ-GT, gamma glutamyl transferase; VL, viral load; IU, international units; HAART, highly active anti retroviral therapy. Values represent mean ± standard deviation.

	HCV mono-infected (Group 1; n = 45)		HIV/HCV co-infected HAART naïve (Group 2; n = 36)		HIV/HCV co-infected HAART received (Group 3: n = 70)		ANOVA *p*-value (adjusted *p)*^$^
	Chronic Group HCVc	Resolved Group HCVr	Chronic Group HIV/HCVc	Resolved Group HIV/HCVr	Chronic Group HIVt/HCVc	Resolved Group HIVt/HCVr	
Cases (N)	24	21	29	7	42	28	
Gender(M/F)	10/11	11/13	14/15	4/3	27/15	13/15	
Age* (y)	53.7 ±8.8	49.5±11.3	55.4±9.3	53.2±6.4	53.03±7.4	50.8±5.2	
HIV genotype	-	-	clade B	clade B	clade B	clade B	
HCV genotype of chronically infected patients							
1b	14	-	7	-	19	-	
2a	7	-	14	-	13	-	
1b and 2a mix	-	-	-	-	9	-	
Unknown genotype	3	-	8	-	1	-	
ALT*(U/L)	58.34±54.68	25.8±17.9	62.1±70.2	38.1±43.6	53.6±57.6	27.9±15.2	3.3E-02(2.3E-01)
Tbil*(U/L)	13.12±6.4	10.2±4.0	13.6±7.1	8.4±1.9	15.8±9.7	11.4±7.1	3.4E-02(2.4E-01)
ALP* (U/L)	23.1±6.9	20.5±6.6	25.6±7.8	22.7±5.2	29.1±9.5	29.4±12.5	2.2E-03(1.5E-02)^%^
γ-GT* (U/L)	57.5±87.2	38.2±24.7	116.7±210.9	27.6±7.1	123.9±141.1	91.3±98.8	7.9E-02(5.5E-01)
HCV VL*[log(IU/ml)]	6±1.3	-	6.7±0.6	-	6.7±1.2	-	1.7E-02(1.2E-01)
HIV VL*[log(copies/ml)]	-	-	3.0±1.1	3.5±1.5	1.9±0.5	2.2±0.9	1.3E-07(9.4E-07)
CD4+T cell count/ml*	851±441	916±349	450±311	417±111	365±243	465±257	1.9E-11(1.3E-10)^%^

^$^ Adjusted *p* value with Bonferroni correction.

^%^*p*<0.05

### Short term HAART treatment has no impact on liver disease progression and HCV viral load

**Fig 1:**
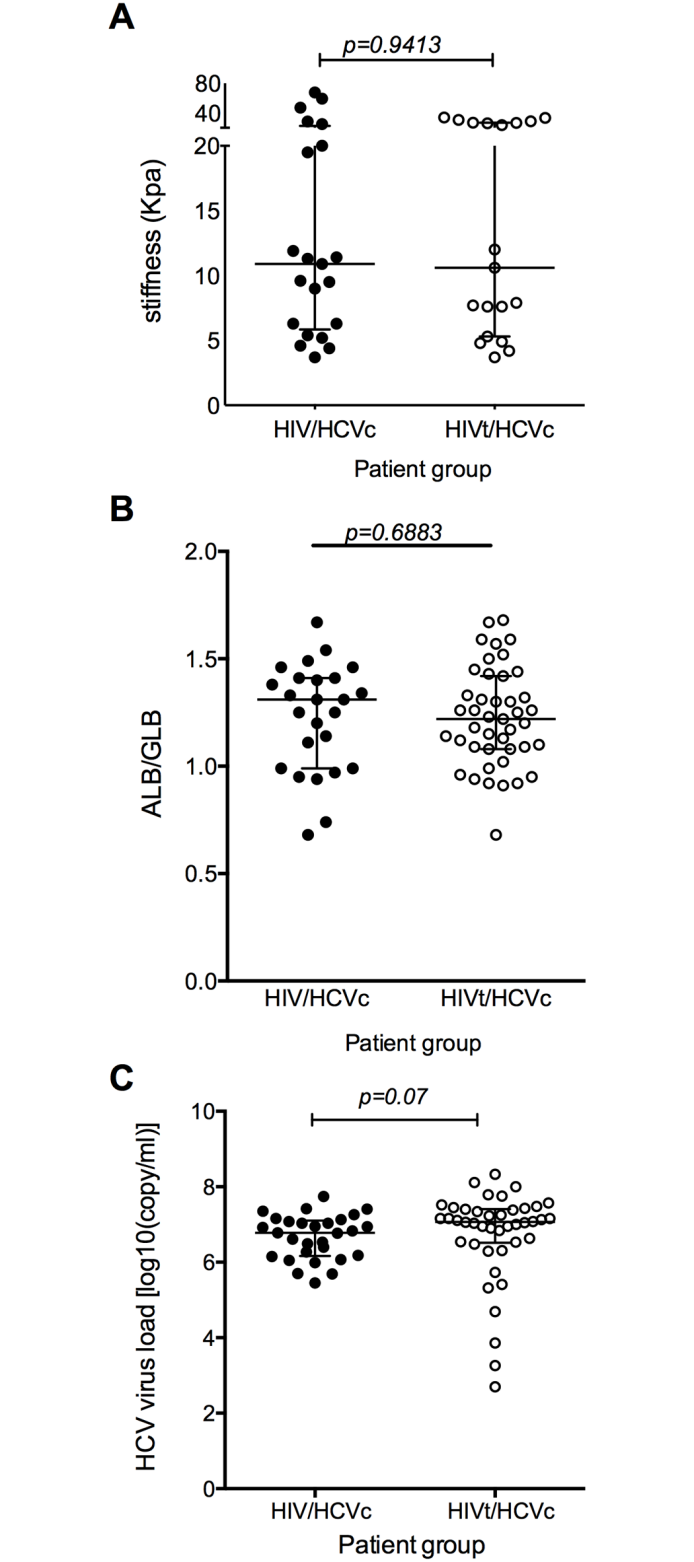
Short term HAART has no effect on the progression of chronic liver disease in HIV/HCV Co-infection group. **A)** The degree of liver fibrosis (Group HIV/HCVc (n = 21) vs Group HIVt/HCVc (n = 19) **B)** ALB/GLB ratio (Group HIV/HCVc (n = 25) vs Group HIVt/HCVc (n = 43) and **C)** HCV virus load (Group HIV/HCVc (n = 29) vs Group HIVt/HCVc (n = 42) were compared between subgroups of HIV/HCV co-infected treatment naive group and HIV/HCV co-infected group that received short term HAART. The *p* values calculated by Mann-Whitney U test. Data are presented as median with interquartile range.

Multiple studies have demonstrated HAART could slow down the liver fibrosis in co-infected individuals [24–26], We first compared the treatment naïve patients in SM cohort with the patients who went on short term HAART (less than two years at the time of the last sample collection). No differences were observed in liver fibrosis(Fig 1A, *p* = 0.9413)), ALB/GLB ratio(Fig 1B, *p* = 0.6883) and HCV viral load (Fig 1C, *p* = 0.07).

### HIV infection contributes to liver disease progression and HCV load

**Fig 2:**
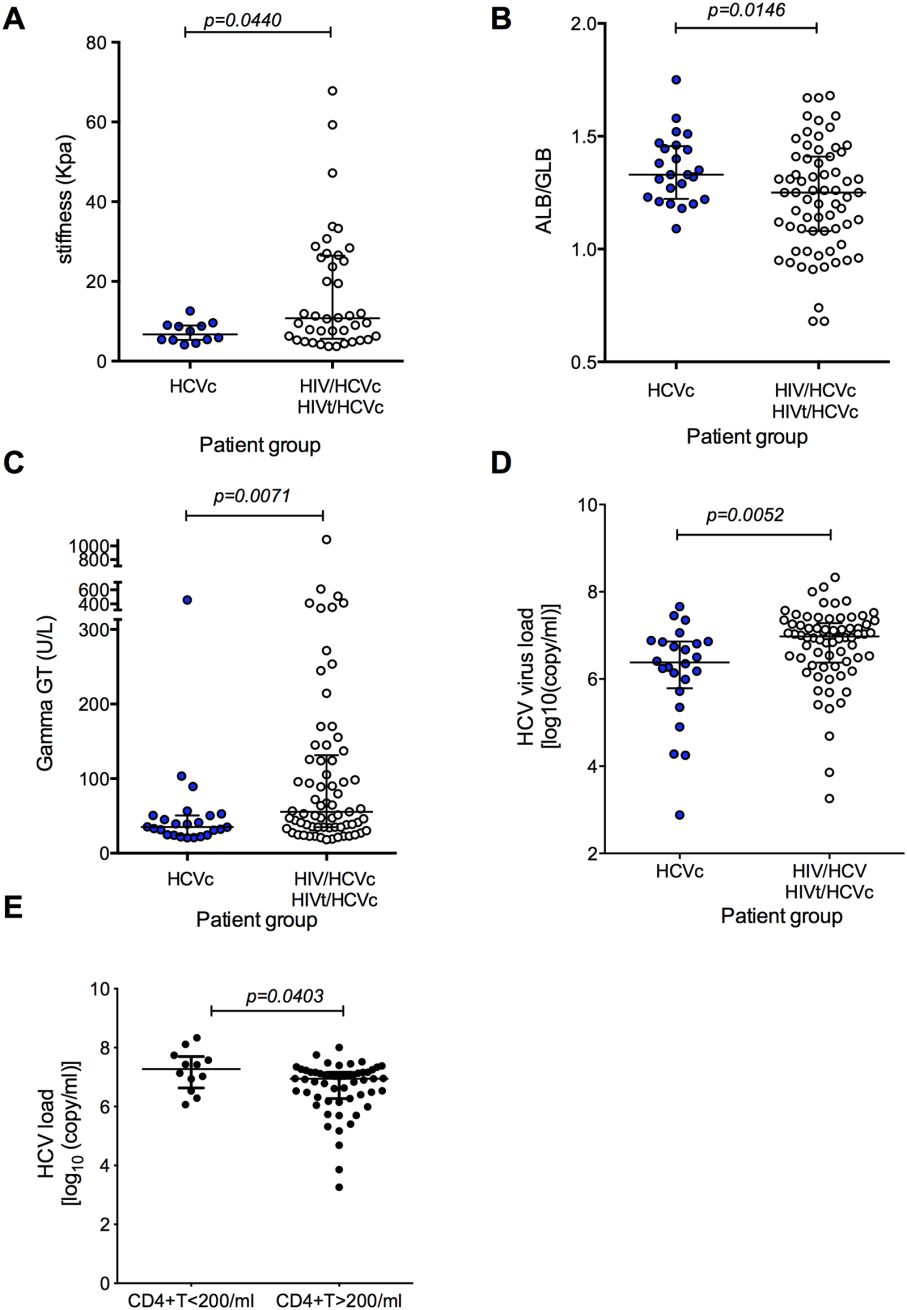
HIV/HCV Co-infection affects the progression of chronic liver disease. **A)** The progression of liver disease was assessed by measuring the degree of liver fibrosis. The progression of liver disease in chronic HIV/HCV co-infected patients (Group HIV/HCVc+ HIVt/HCVc (n = 40)) was faster than chronic HCV mono-infected patients (Group HCVc (n = 12)). **B)** The ALB/GLB ratio was higher in Group HCVc (n = 24) than chronic HIV/HCV co-infected patients (Group HIV/HCVc + HIVt/HCVc (n = 67)). **C)** Gamma GT levels were higher in Group HIV/HCV co-infected patients (Group HIV/HCVc + HIVt/HCVc (n = 69) than HCVc (n = 24). **D)** HCV virus load was lower in Group HCVc (n = 24) than chronic HIV/HCV co-infected patients (Group HIV/HCVc +HIVt/HCVc (n = 70)). **E)** A higher HCV viral loads were observed in patients with low CD4^+^ T cell counts in HIV/HCV Co-infection (CD4+T<200 n = 12, CD4+T>200 n = 55). The *p* values calculated by Mann-Whitney U test. Data are presented as median with interquartile range.

We next noted significantly higher liver fibrosis indices (Fig 2A, *p* = 0.044), lower mean albumin to globulin ratio (Fig 2B, *p* = 0.0146), and higher serum gamma-GT levels (Fig 2C, *p* = 0.0071) in HIV/HCV co-infected (HIV/HCVc + HIVt/HCVc) subjects compared to HCV mono-infected subjects. For this analysis the patient groups HIV/HCVc and HIVt/HCVc were pooled together to increase the statistical power, as short term HAART had no impact on liver disease progression (discussed above). Importantly, these results demonstrate that HIV contributes to an increased hepatic damage in patients co-infected with HCV.

We next assessed whether HCV infection modulated HIV progression by comparing the HIV viral load and CD4+ T cell count in HAART naïve HIV/HCV co-infected patients (HIV/HCVc vs HIV/HCVr). We observed no apparent difference between HIV viral loads (S3A Fig) or CD4^+^ T cell counts (S3B Fig) in patients with chronic(HIV/HCVc) versus resolved HCV(HIV/HCVr).

However, we found HCV viral load was significantly higher in HIV/HCV co-infected compared to HCV mono-infected (Fig 2D, *p* = 0.0052). We evaluated whether the clinical stage of HIV has an impact on HCV viral load by stratifying co-infected patients with respect to their HIV viral copy number and CD4^+^ T cell count. Of note, we did not observe any association between HIV load and HCV RNA levels (data not shown), however, a higher HCV viral loads were observed in patients with low CD4^+^ T cell counts (Fig 2E, *p* = 0.0403).

### A role for HCV specific T cell responses in controlling chronic HCV replication

**Fig 3:**
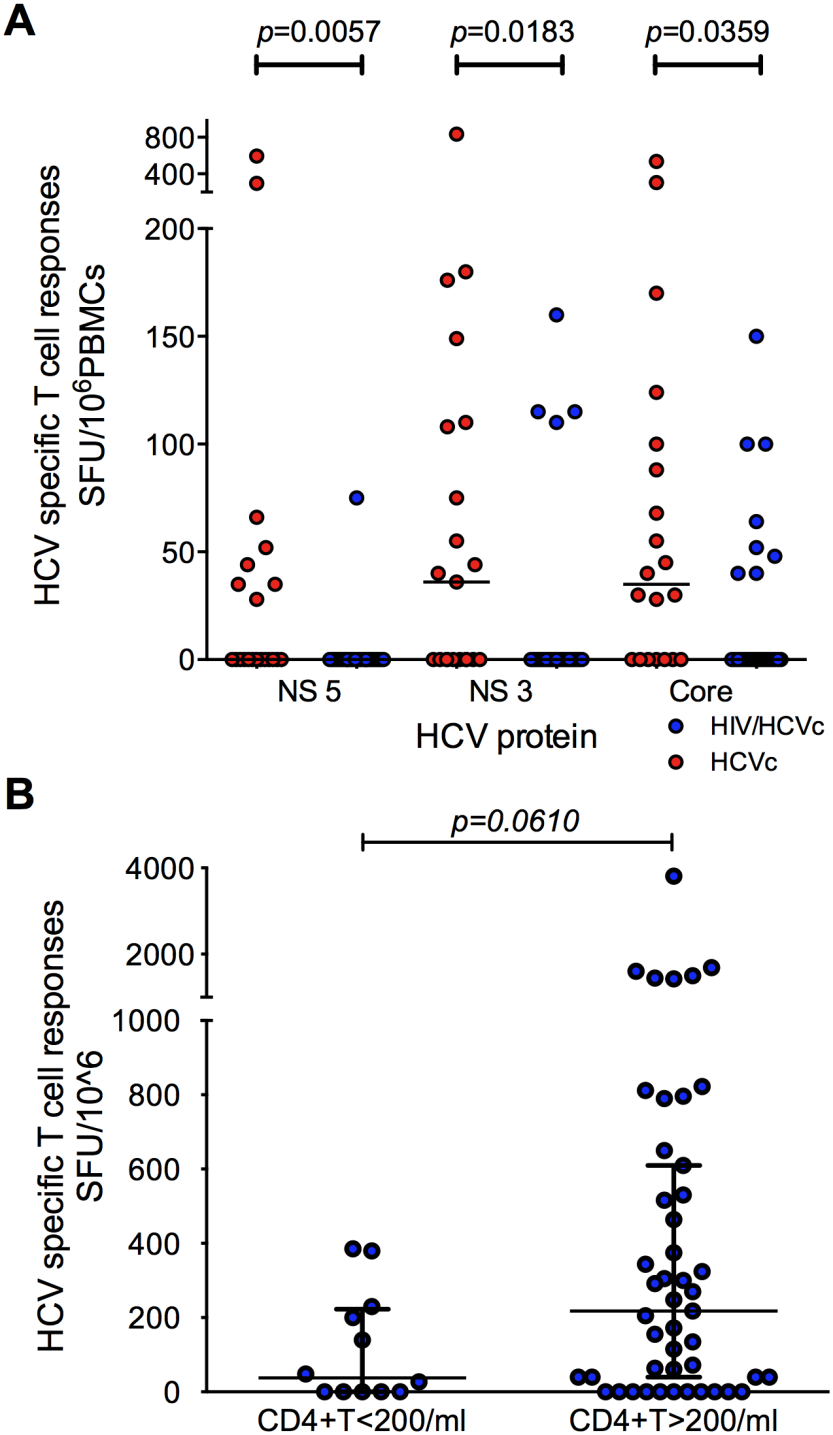
HCV specific T cell responses are important in the control of HCV viral load and are higher in HCV mono infected patients. **A)** HCV core and NS protein specific T cell responses were compared between a sub group of HCV mono-infected (Group HCVc (n = 21) and HAART naïve HIV/HCV co-infected patients (group HIV/HCVc (n = 24)). Median is shown. **B)** HCV specific T cell responses were compared between CD4>200 and CD4<200 in HCV/HIV co-infection group (n = 60). Data are presented as median with interquartile range. The *p* values calculated by Mann-Whitney U test.

To test the hypothesis that HIV-1 infection diminishes the frequency of T cell responses against HCV, we compared the total T cell responses in HCV mono-infected and HAART naïve HIV/HCV co-infected groups by ELISPOT assay using overlapping 18mer peptide pools spanning the entire HCV proteome. Representative examples of T cell responses to HCV peptides in our *ex-vivo* ELISPOT assays are shown in S4 Fig. We observed a significantly higher frequency of IFNγ producing HCV-specific responses to core (*p* = 0.0359), NS3 (*p* = 0.0183) and NS5 (*p* = 0.0057) in HCV mono-infected patients compared to co-infected patients (Fig 3A). There is a tendency, although not statistically significant, towards a better HCV-specific T cell response in patients with CD4 counts >200, suggesting that the lack of CD4^+^ T cell help in co-infection likely has an impact on HCV specific Immunity (Fig 3B).

### HIV co-infection primarily impairs HCV specific CD4^+^ T cell responses

**Fig 4:**
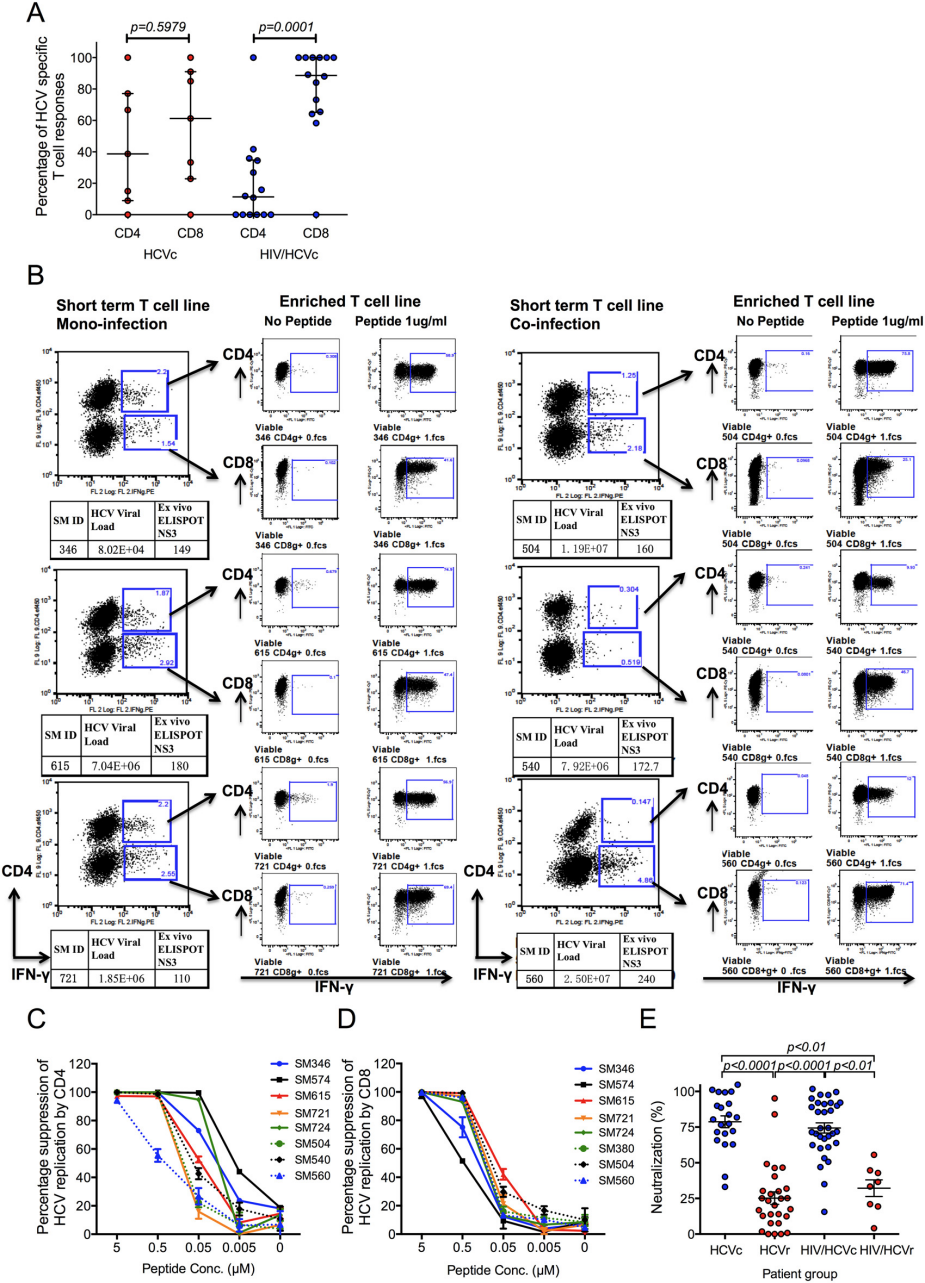
**A)** Reduced percentage of CD4+ specific T cell responses in HIV co-infected patients. The percentage of HCV specific CD4+ or CD8+ T cell responses were measured in a subset of HIV/HCV co-infected (n = 14) and HCV mono-infected (n = 7) individuals following *ex-vivo* stimulation of PBMC and IFNγ ELISPOT assay after CD8+ T cell depletion (See Materials and methods and S5 Fig). The *p* values calculated by Mann-Whitney U test. Data are presented as median with interquartile range. **B)** Representative data from HCV NS3 peptide stimulated enriched short-term T cell lines from three patients each of HCV mono-infected (Group HCVc) and HIV/HCV co-infected (group HIV/HCVc) to demonstrate HCV specific IFNγ response. CD4^+^ and CD8^+^ dependency of IFNγ responses were analysed after flow cytometry based sorting. **C and D** The antiviral activity of enriched HCV NS3 specific T cells. HCV suppression by peptide stimulated **(C)** bulk CD4+ T cells (n = 4 replicates) and (**D)** bulk CD8+ T cells (n = 4 replicates). Solid lines represent HCV mono-infected patients; dash lines represent HIV/HCV co-infected patients. The percentage suppression was calculated by measuring luciferase activity after 48 hours of co-culture of enriched HCV NS3 specific T cells with HLA-A2 transfected Huh7.5 HCV replicon cells. Bars represent mean + SD. **E)** HIV has no impact on HCV neutralizing antibodies. Serum from a subgroup of patients co-infected with HCV and HIV (group HIV/HCVc, HIV/HCVr, HIVt/HCVc & HIVt/HCVr, n = 40) and patients with HCV mono-infection (group HCVc & HCVr, n = 49) were used to neutralize the infectivity of H77 HCVpp. The percentage of neutralization by patient serum was determined for patients positive of HCV viral load (blue symbols) and negative for HCV viral load (red symbols). The data were analyzed by Kruskal-Wallis test, and followed with Dunn’s multiple comparisons test. Each point is representative of the mean of one patient from two independent experiments. Data are presented as mean with SEM.

To better understand the role of HIV on HCV specific adaptive immune responses we measured functional CD4^+^ and CD8^+^ T cells in a subset of HCV mono-infected and HAART naïve HIV co-infected individuals using an IFNγ ELISPOT assay with CD4 or CD8 depletion [21](Fig 4A and 4B and S5 Fig). The relative proportion of CD8^+^ specific T cell responses from total T cell responses were significantly higher in HIV/HCV co-infected individuals compared to HCV mono-infected individuals (*p* = 0.0001 vs *p* = 0.5979, Fig 4A). As expected we noted reduced CD4^+^ specific T cell responses in HIV co-infected patients, most likely secondary to HIV mediated CD4^+^ depletion (Fig 4A and S6 Fig). To confirm the CD4^+^ or CD8^+^ dependency of the HCV-specific responses we generated short-term T cell lines from HCV mono- and HIV co-infected subjects. Strong CD4^+^ and CD8^+^ T cell responses were maintained after peptide stimulation of STLs (Fig 4B). Finally, we measured the anti-viral activity of HCV-specific CD4^+^ and CD8^+^ T cell lines in the well characterised HCV-luciferase reporter system [27]. HCV NS3 specific CD4^+^ and CD8^+^ T cells were enriched by sorting for IFNγ expressing cells following peptide stimulation and the conditioned media harvested to measure anti-viral activity. Importantly, peptide stimulated bulk CD4^+^ T cells inhibited HCV replication to comparable levels as CD8^+^ T cells (Fig 4C and 4D).

### HIV has no effect on HCV neutralizing antibody production

Finally, we analysed the effect of HIV co-infection on HCV specific neutralizing antibody responses in chronic and resolved infection. As expected, we observed a statistically significant higher level of neutralization in chronic versus resolved HCV infection (Fig 4E). However, no differences were observed between HCV mono-infected and HIV/HCV co-infected patients (Fig 4E).

## Discussion

Access to this unique HCV mono and HIV/HCV co-infected plasma donor cohort provided us with an opportunity to study the role of HIV on HCV-specific immunity and its association with liver disease progression. Our results provide important insights into the immunobiology of HCV and the impact of HIV co-infection. We confirm that HIV co-infection leads to an accelerated liver disease progression [3, 5], short treatment of HAART seemed having no effect on liver fibrosis and HCV viral load. The mechanisms underlying this observation are likely to be multi-factorial, the lower HCV-specific T cell responses observed in HIV co-infection as a consequence of decreased CD4^+^ T cells are likely to have contributed. Indeed, we found that the frequency of HCV specific T cell responses in particular CD4^+^ T cells correlated with CD4^+^ T cell counts. Exhaustion of HCV-specific T cells in co-infected individuals that has been described before is another factor that is likely to have contributed [28]. Higher HCV load and lower T cell responses were observed in patients with low CD4 counts, however, we did not observe any significant differences using correlation analysis, which could due to insufficient number of patients studied. Importantly, we noted an inverse association between HCV specific T cell responses and HCV load and reduced fibrosis in chronic HCV infection. This protective effect is linked to a significantly higher virus-specific T cell responses targeting the HCV structural proteins NS3 and NS5 in HCV mono- compared to HIV co-infected patients. These results are in line with previous reports showing stronger HCV NS3 and NS5 specific T cell responses in subjects who had resolved HCV infection compared to chronically infected subjects [29, 30].

HIV co-infection has been reported having a negative impact on HCV-specific CD8^+^ T cell responses [31, 32]. We observed much greater impact on CD4^+^ than CD8^+^, but we also observed overall tendency of lower T cell responses (both CD4^+^ and CD8^+^) in patients with CD4<200, indicating CD8^+^ T cells were also affected and likely associated with lower CD4^+^ counts in this cohort, which agrees with previous report.

Interestingly, we found that despite their diminished frequency and function, *ex-vivo* expanded HCV-specific CD4^+^ T cells controlled virus replication as efficiently as HCV specific CD8^+^ T cells in both mono and co-infected. These results may have important implications for immunotherapy as they suggest that CD4^+^ T cells can rapidly regain their antiviral activity after *in-vitro* expansion without further specific interventions, such as blockade of specific inhibitory receptors, e.g.PD-1, supporting adoptive T cell therapy.

It is worth noting that most of the co-infected subjects in the SM cohort were likely infected first with HCV or co-infected at the same time, since epidemiological studies show blood born HCV transmission to be at least 10 times higher than HIV [3, 33]. The presence of HCV mono and HIV/HCV co-infected individuals but no HIV mono-infections among plasma donors in this cohort supports the higher risk of transmission of HCV. Furthermore, the higher rate of HCV persistence in the co-infected group may be explained by recurrent HCV infection after HIV co-infection [3, 34]. This scenario is further supported by the individuals co-infected with HCV 1b and 2a subtypes (Table 1).

HCV-specific T cells immune responses were detected in the majority of chronic HCV subjects in the cohort, in contrast to previous reports suggesting minimal T cell responses in chronic HCV infection [32, 35–37]. These differences may be attributable to the use of freshly isolated PBMC for all experiments or to the use of HCV peptides spanning genotypes 1b and 2a that represent HCV strains present in our cohort [32, 35–37]. Finally, differences in host genetic background between Chinese and western cohorts, and also the infecting virus genotype may account for the observed differences.

In contrast to the significant effect of HIV on CD4^+^ T cell responses, we failed to observe any effect on HCV specific neutralizing antibody levels. HCV and HIV are the most genetically diverse viruses that rapidly change their antigenic profiles, constituting a major challenge for vaccines that elicit cross-reactive neutralizing antibodies [38]. Nevertheless, neutralizing antibodies have proven to be important in controlling both HIV and HCV infection as well as the chimpanzee model [39, 40]. HIV is known to deplete CD4^+^ T cells, an important regulator in B cell activation and class switching leading to global decline in neutralising antibody [41]. Thus, it is striking that we did not find a significant impact of HIV on HCV neutralizing antibodies. Our results suggest that on-going HCV replication is the primary determinant maintaining neutralizing antibody responses [42, 43].

Taken together, we found that HIV infection contributes to progressive liver disease in the context of HCV co-infection, most likely by modulating HCV specific T cell responses in particular CD4^+^ T cells but not HCV specific neutralizing antibody responses. HCV specific T cell responses targeting the non-structural proteins associate with low HCV RNA levels, further supporting a protective role for HCV-specific T cells.

## Supporting Information

S1 Table HCV peptide pool design. (PDF)

S1 Fig Analysis between HCV 2a subtype core specific T cell responses and 1b core specific T cell response. (PDF)

S2 Fig Gating strategy of sorted short term T cell lines. (PDF)

S3 Fig HCV has no impact on HIV progression. The impact of chronic HCV on HIV progression in HIV/HCV chronically infected HAART naïve patients (group HIV/HCVc vs HIV/HCVr, n = 35) were assessed by comparing HIV viral load (A) and CD4 counts stratified by HCV clearance (B). Data represent mean+ SD, p as calculated by Mann Whitney test.
(PDF)

S4 Fig Representative data of IFNγ ELISPOT responses from a HCV mono-infected patient and a HIV/HCV co-infected patient. (PDF)

S5 Fig Representative IFNγ ELISPOT responses to HCV peptides before and after depletion of CD8+ T cells. (PDF)

S6 Fig The CD4+T cell count was significantly lower in HIV/HCV co-infection group compared to mono HCV infection group. (PDF)
