# Supplementary material for: A Comprehensive Analysis of the Impact of HIV on HCV Immune Responses and Its Association with Liver Disease Progression in a Unique Plasma Donor Cohort
Source: PLoS One. 2016 Jul 25;11(7):e0158037. doi: 10.1371/journal.pone.0158037 (PMC4959707; doi:10.1371/journal.pone.0158037)
Supplement: S1 Table — (PDF) [file pone.0158037.s001.pdf]

S1 Table: HCV peptide pool design

Peptides pool	Number of peptides
core	28
NS2	32
NS3-1	50
NS3-2	48
NS4A	7
NS4B	40
NS5A-1	35
NS5A-2	36
NS5B-1	45
NS5B-2	45
E1	28
E2-1	30
E2-2	25
p7	8
ARFP	22
TOTAL	479
