# Supplementary material for: A Comprehensive Analysis of the Impact of HIV on HCV Immune Responses and Its Association with Liver Disease Progression in a Unique Plasma Donor Cohort
Source: PLoS One. 2016 Jul 25;11(7):e0158037. doi: 10.1371/journal.pone.0158037 (PMC4959707; doi:10.1371/journal.pone.0158037)

S3 Fig.: HCV has no impact on HIV progression. The impact of chronic HCV on HIV progression in HIV/HCV chronically infected HAART naïve patients (group HIV/HCVc vs HIV/HCVr, n=35) were assessed by comparing HIV viral load (A) and CD4 counts stratified by HCV clearance (B). Data represent mean+ SD, *p* as calculated by Mann Whitney test.

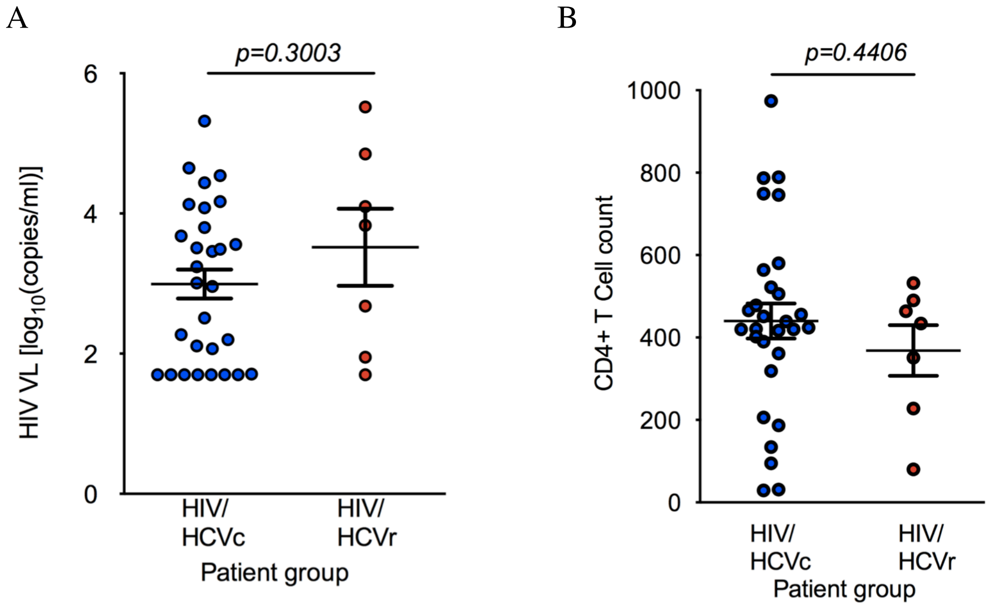

Supplement: S3 Fig — The impact of chronic HCV on HIV progression in HIV/HCV chronically infected HAART naïve patients (group HIV/HCVc vs HIV/HCVr, n = 35) were assessed by comparing HIV viral load (A) and CD4 counts stratified by HCV clearance (B). Data represent mean+ SD, p as calculated by Mann Whitney test. (PDF) [file pone.0158037.s004.pdf]
