# Supplementary material for: A Comprehensive Analysis of the Impact of HIV on HCV Immune Responses and Its Association with Liver Disease Progression in a Unique Plasma Donor Cohort
Source: PLoS One. 2016 Jul 25;11(7):e0158037. doi: 10.1371/journal.pone.0158037 (PMC4959707; doi:10.1371/journal.pone.0158037)

S4 Fig.: Representative data of IFNγ ELISPOT responses from a HCV mono-infected patient and a HIV/HCV co-infected patient

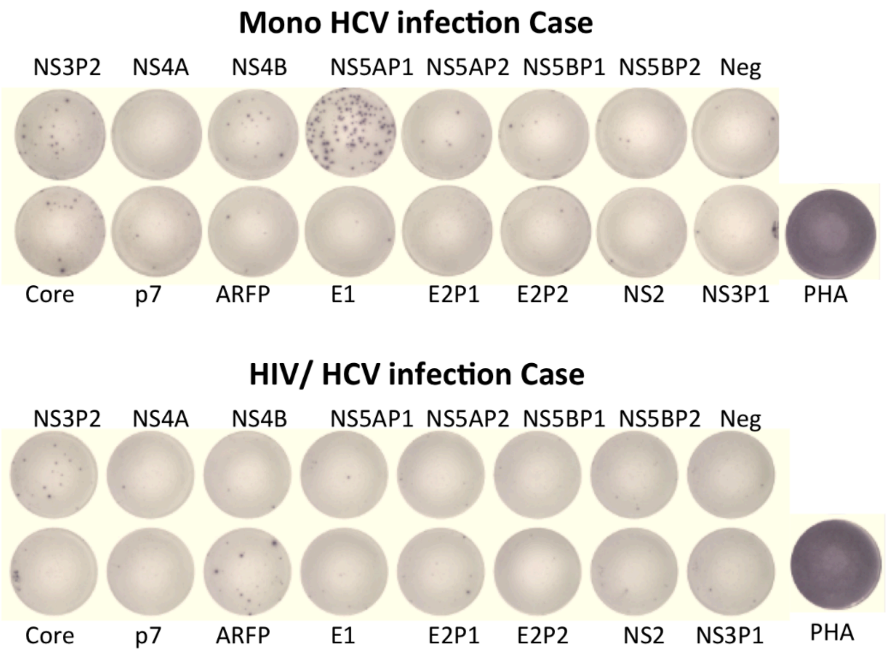

Supplement: S4 Fig — (PDF) [file pone.0158037.s005.pdf]
