# Supplementary material for: A Comprehensive Analysis of the Impact of HIV on HCV Immune Responses and Its Association with Liver Disease Progression in a Unique Plasma Donor Cohort
Source: PLoS One. 2016 Jul 25;11(7):e0158037. doi: 10.1371/journal.pone.0158037 (PMC4959707; doi:10.1371/journal.pone.0158037)

S5 Fig.: Representative IFNγ ELISPOT responses to HCV peptides before and after depletion of CD8+ T cells.

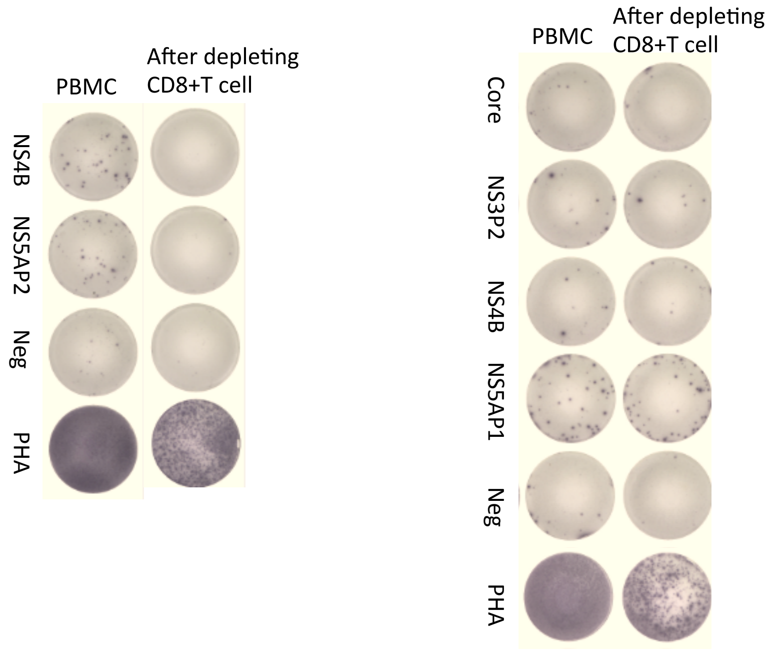

Supplement: S5 Fig — (PDF) [file pone.0158037.s006.pdf]
