# Supplementary material for: A Comprehensive Analysis of the Impact of HIV on HCV Immune Responses and Its Association with Liver Disease Progression in a Unique Plasma Donor Cohort
Source: PLoS One. 2016 Jul 25;11(7):e0158037. doi: 10.1371/journal.pone.0158037 (PMC4959707; doi:10.1371/journal.pone.0158037)

S6 Fig.: The CD4+T cell count was significantly lower in HIV/HCV co-infection group compared to mono HCV infection group. Data represent mean+ SD, *p* as calculated by Mann Whitney test.

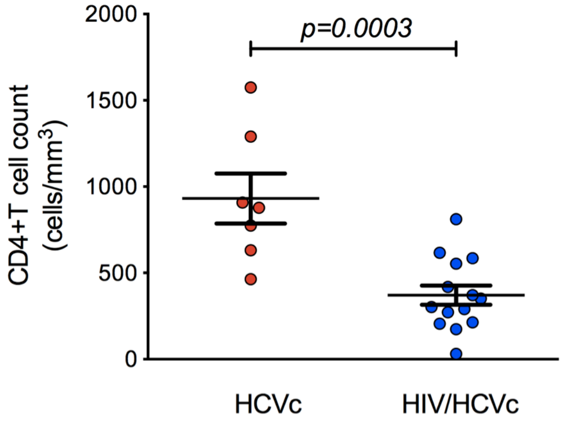

Supplement: S6 Fig — (PDF) [file pone.0158037.s007.pdf]
